# RNA editing facilitates the enhanced production of neoantigens during the simultaneous administration of oxaliplatin and radiotherapy in colorectal cancer

**DOI:** 10.1038/s41598-022-17773-0

**Published:** 2022-08-08

**Authors:** Yasuhiro Komatsu, Kunitoshi Shigeyasu, Shuya Yano, Sho Takeda, Kazutaka Takahashi, Nanako Hata, Hibiki Umeda, Kazuhiro Yoshida, Yoshiko Mori, Kazuya Yasui, Ryuichi Yoshida, Yoshitaka Kondo, Hiroyuki Kishimoto, Fuminori Teraishi, Yuzo Umeda, Shunsuke Kagawa, Hiroyuki Michiue, Hiroshi Tazawa, Ajay Goel, Toshiyoshi Fujiwara

**Affiliations:** 1 Department of Gastroenterological Surgery, Okayama University Graduate School of Medicine, Dentistry, and Pharmaceutical Sciences, 2-5-1 Shikata-cho, Kita-ku, Okayama, 700-8558 Japan; 2 Department of Molecular Diagnostics and Experimental Therapeutics, Beckman Research Institute, City of Hope Biomedical Research Center, Monrovia, CA USA; 3 City of Hope Comprehensive Cancer Center, Duarte, CA USA; 4 Neutron Therapy Research Center, Okayama University, Okayama, Japan

**Keywords:** Colorectal cancer, Cancer epigenetics

## Abstract

Most cases of colorectal cancers (CRCs) are microsatellite stable (MSS), which frequently demonstrate lower response rates to immune checkpoint inhibitors (ICIs). RNA editing produces neoantigens by altering amino acid sequences. In this study, RNA editing was induced artificially by chemoradiation therapy (CRT) to generate neoantigens in MSS CRCs. Altogether, 543 CRC specimens were systematically analyzed, and the expression pattern of ADAR1 was investigated. In vitro and in vivo experiments were also performed. The RNA editing enzyme ADAR1 was upregulated in microsatellite instability–high CRCs, leading to their high affinity for ICIs. Although ADAR1 expression was low in MSS CRC, CRT including oxaliplatin (OX) treatment upregulated RNA editing levels by inducing ADAR1. Immunohistochemistry analyses showed the upregulation of ADAR1 in patients with CRC treated with CAPOX (capecitabine + OX) radiation therapy relative to ADAR1 expression in patients with CRC treated only by surgery (p < 0.001). Compared with other regimens, CRT with OX effectively induced RNA editing in MSS CRC cell lines (HT29 and Caco2, p < 0.001) via the induction of type 1 interferon-triggered ADAR1 expression. CRT with OX promoted the RNA editing of cyclin I, a neoantigen candidate. Neoantigens can be artificially induced by RNA editing via an OX–CRT regimen. CRT can promote proteomic diversity via RNA editing.

## Introduction

Colorectal cancer (CRC) is the second most common cause of cancer-related deaths in the USA^1^. However, accumulating evidence from recent decades suggests that molecular targeted therapy can prolong the survival of patients with CRC. Immune checkpoint inhibitors (ICIs) have emerged as “game changers” in cancer therapy; in CRC treatment, ICIs respond mainly to mismatch repair-deficient CRCs, which exhibit microsatellite instability–high (MSI-H) tumors. However, nearly 85% of CRCs are microsatellite stable (MSS)^2^ and frequently demonstrate lower response rates to ICIs. One of the reasons for this phenomenon is differences in the tumor mutation burden (TMB). MSS CRCs have a TMB lower than that of MSI-H CRCs; thus, cytotoxic T lymphocytes are unable to target neoantigens (a protein that forms on cancer cells when certain mutations occur in tumor DNA), resulting in “cold,” or non-immunogenic tumors (i.e., those containing few infiltrating T cells)^3^. Recent research efforts have aimed to identify mechanisms by which cold tumors can be changed to “hot” tumors (i.e., those characterized by T cell infiltration)^3^. In this context, RNA editing has emerged as one of the crucial processes that allows increased production of neoantigens in MSS CRCs, thus artificially transforming poorly immunogenic tumors into highly immunogenic and hot tumors.

RNA editing is a recently identified epigenetic mechanism that produces neoantigens by altering amino acid sequences via RNA modification^4,5^. A comprehensive RNA editing neoantigen profile analysis of 12 cancer types was recently published^6^. Adenosine-to-inosine RNA editing is mediated by a group of enzymes known as adenosine deaminases that act on RNA (ADARs)^7^. Although RNA editing is a mechanism whereby neoantigens can be produced in cancer cells^8^, leading to an improved response to ICIs^6^, the process by which RNA editing is regulated and produces neoantigens remains unclear.

In this study, how RNA editing can be controlled to transform immunogenically cold tumors into hot tumors was illustrated. Whether RNA editing was induced by existing treatments, such as chemotherapy and radiotherapy, using clinical specimens and an in-vitro experimental model was determined. Chemoradiation therapy (CRT), including oxaliplatin (OX), was shown to upregulate RNA editing levels by inducing the RNA editing enzyme ADAR1, which is triggered by type 1 interferon. Of note, CRT with OX promoted RNA editing of cyclin I (CCNI), which was previously reported to be a candidate of neoantigens induced by RNA editing in patients with melanoma^8^. Overall, our results suggest that neoantigens can be artificially induced by RNA editing activated by an OX–CRT regimen; this epigenetic modification may also compensate for lower TMB in MSS CRC during immunotherapy. Thus, our study will provide a novel molecular basis for CRT and ICI combination therapy in CRC.

## Results

### ADAR1 is upregulated in CMS1 and MSI CRCs

Whether immune-related markers are associated with any consensus molecular subtype (CMS) subgroups in CRC, a classification based on heterogeneity at the gene expression level, was assessed^9^. MSI CRCs are classified as CMS1. The relationship between the MSI status, CMS classification, and RNA editing as a potential new source of neoantigens for immunotherapy was first analyzed.

**Figure 1:**
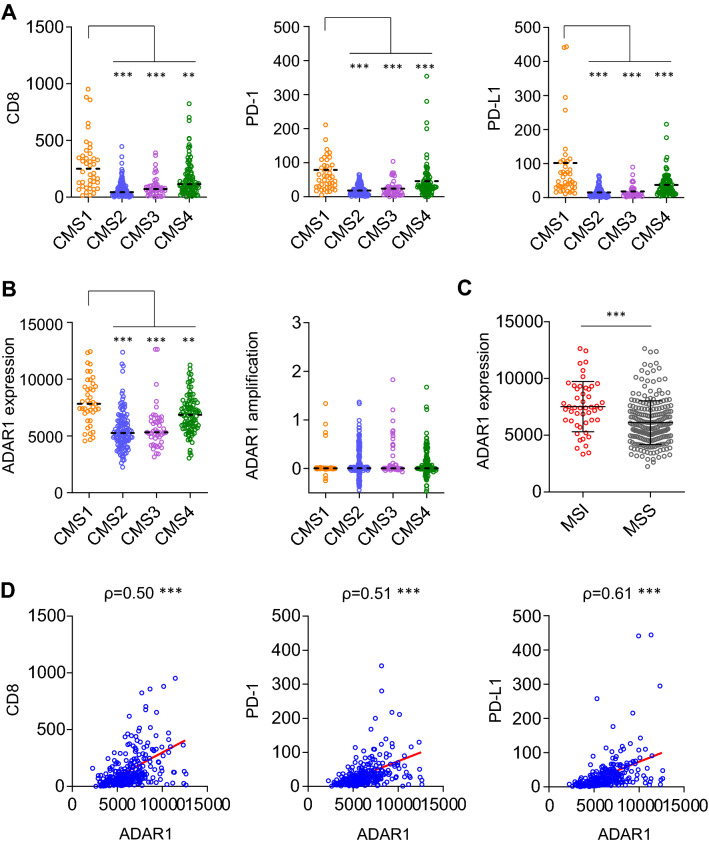
ADAR1 is upregulated in CMS1 and MSI CRCs. (**A**) Expression analysis of CD8, PD-1, and PD-L1 in CRC was performed using the TCGA database. Vertical axis shows RSEM (batch normalized from Illumina HiSeq_RNASeqV2). Patients were classified as CMS1–4 according to the consensus molecular subtypes. CMS1 has a strong immune activation effect, showing upregulation of CD8, the surface marker of cytotoxic T cells (p < 0.01). Additionally, PD-1 and PD-L1 (both p < 0.001) are upregulated in CMS1 CRCs. Steel test. (**B**) ADAR1 was upregulated to a larger extent in CMS1 CRCs compared with its expression in other subtypes (p < 0.01). However, amplification levels of ADAR1 did not differ between each subtype. Steel test. (**C**) ADAR1 was upregulated in MSI CRCs compared with its expression in MSS CRCs (p < 0.001). Wilcoxon’s signed rank test. (**D**) ADAR1 showed a positive correlation with CD8 (ρ = 0.50, p < 0.001), PD-1 (ρ = 0.51, p < 0.001), and PD-L1 (ρ = 0.61, p < 0.001). **p < 0.01; ***p < 0.001.

The patients with CRC classified as CMS1 showed strong immune activation, with upregulation of CD8, the surface marker of cytotoxic T cells (p < 0.01; Fig. 1A). In addition, programmed cell death 1 (PD-1) and programmed cell death ligand 1 (PD-L1) (both p < 0.001) were upregulated in CMS1 CRCs. PD-1 and PD-L1 are both known predictive markers of cancer immunotherapy, suggesting that CMS1 CRCs have high affinity to ICIs. This is primarily by virtue of which MSI CRCs are abundant in neoantigens and are classified as CMS1^10^.

Next, the expression pattern of the RNA editing enzyme ADAR1 was assessed because ADAR1 can produce neoantigens through RNA editing^8^. ADAR1 was upregulated to a larger extent in CMS1 CRCs compared with other subtypes (p < 0.01; Fig. 1B). However, amplification levels of ADAR1 did not change between subtypes, suggesting that the cause of ADAR1 upregulation in CMS1 CRCs was not genetic amplification but rather transcriptional activation. Furthermore, ADAR1 was upregulated in MSI CRCs relative to its expression in MSS CRCs (p < 0.001; Fig. 1C). Thus, ADAR1 appears to be upregulated in MSI or CMS1 CRCs, resulting in high affinity for ICIs, likely because RNA editing produces neoantigens in a posttranscriptional manner^8^. MSI CRCs possess two distinct advantages for cancer immunotherapy: high TMB based on deficient mismatch repair and epigenetic diversity based on high RNA editing activity. ADAR1 showed a positive correlation with CD8 (ρ = 0.50, p < 0.001), PD-1 (ρ = 0.51, p < 0.001), and PD-L1 (ρ = 0.61, p < 0.001; Fig. 1D), providing evidence that the upregulation of ADAR1 may be associated with the immunological response to CRC. Indeed, increased expression of ADAR1 has previously been associated with increased abundance of tumor-infiltrating lymphocytes in patients with breast cancer, which is in agreement with the present findings^11^. Patients with high RNA editing tumors treated using ICI have shown better prognosis in melanoma^6^. Altogether, these results show that artificial upregulation of ADAR1 followed by promotion of RNA editing and production of neoantigens may improve the response to ICIs in MSS CRCs.

### ADAR1 is upregulated in CRCs treated with CRT

Bioinformatics analysis revealed that increased expression of the RNA editing enzyme ADAR1 was associated with an increased immunogenic response. Because RNA editing can generate neoantigens, including edited CCNI^8^, the artificial upregulation of ADAR1 could contribute to improving cancer immunotherapy.

In this context, CRT was focused upon because it can generate type 1 interferon response, which promotes the expression of the RNA editing enzyme ADAR1. Indeed, both chemotherapy and radiotherapy are reported as activators of cancer immunotherapy^12,13^.

**Figure 2:**
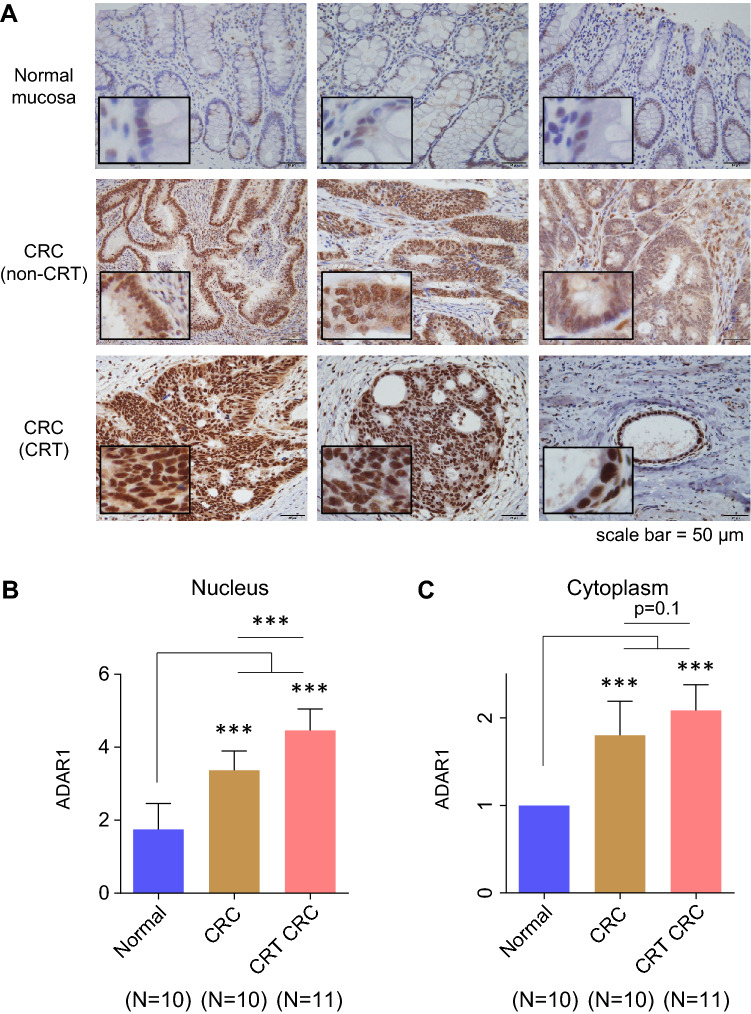
ADAR1 is upregulated in CRCs treated with CRT. (**A**) IHC analysis revealed strong staining for ADAR1 in CRCs treated with CRT. (**B**) ADAR1 was upregulated in the nucleus of CRT-CRCs relative to the normal mucosa (p < 0.001) and in CRCs without CRT (p < 0.001). (**C**) ADAR1 was also upregulated in the cytoplasm of CRT-CRCs compared with normal mucosa (p < 0.001) or CRCs without neoadjuvant therapy (p < 0.001). Wilcoxon’s signed-rank test. ***p < 0.001.

First, immunohistochemical (IHC) analysis was performed in clinical specimens. Because ADAR1 is expressed in both the nucleus and cytoplasm, ADAR1 expression levels were observed separately in each compartment. IHC analysis revealed strong staining for ADAR1 in CRCs treated with CRT (CAPOX-RT: capecitabine + OX + RT) (Fig. 2A). ADAR1 was upregulated in the nuclei of CRT-treated CRCs compared with the normal mucosa or untreated CRCs (both p < 0.001; Fig. 2B). ADAR1 was also upregulated in the cytoplasm of CRT-treated CRCs compared with normal mucosa (p < 0.001) but not with untreated CRCs (Fig. 2C). Collectively, these results indicate that only nuclear ADAR1 was preferentially upregulated in CRT (CAPOX-RT)-treated CRCs compared with untreated CRCs. From the viewpoint of the so-called PANoptosis^14^, wherein cell death occurs while presenting cancer antigens, the location of ADAR1 is problematic. Cytoplasmic ADAR1 suppresses PANoptosis and tumor immunity consequently^15^. Despite the increase in ADAR1 expression by CRT, a noteworthy elevation is noted only in the nucleus. However, during the production of neoantigens via RNA editing in the nucleus, this expression is not sufficient for the inhibition of PANoptosis. A positive correlation was also found between the expression level of ADAR1 in the nucleus and the surrounding lymphocyte population in CRT-treated CRCs (ρ = 0.74, p < 0.05, Supplementary Fig. 1). This suggests that ADAR1 expression in the nucleus has an immunogenic effect in CRT cases.

### ADAR1 expression and CCNI editing are promoted by chemotherapy, radiation, and CRT in CRC cells

**Figure 3:**
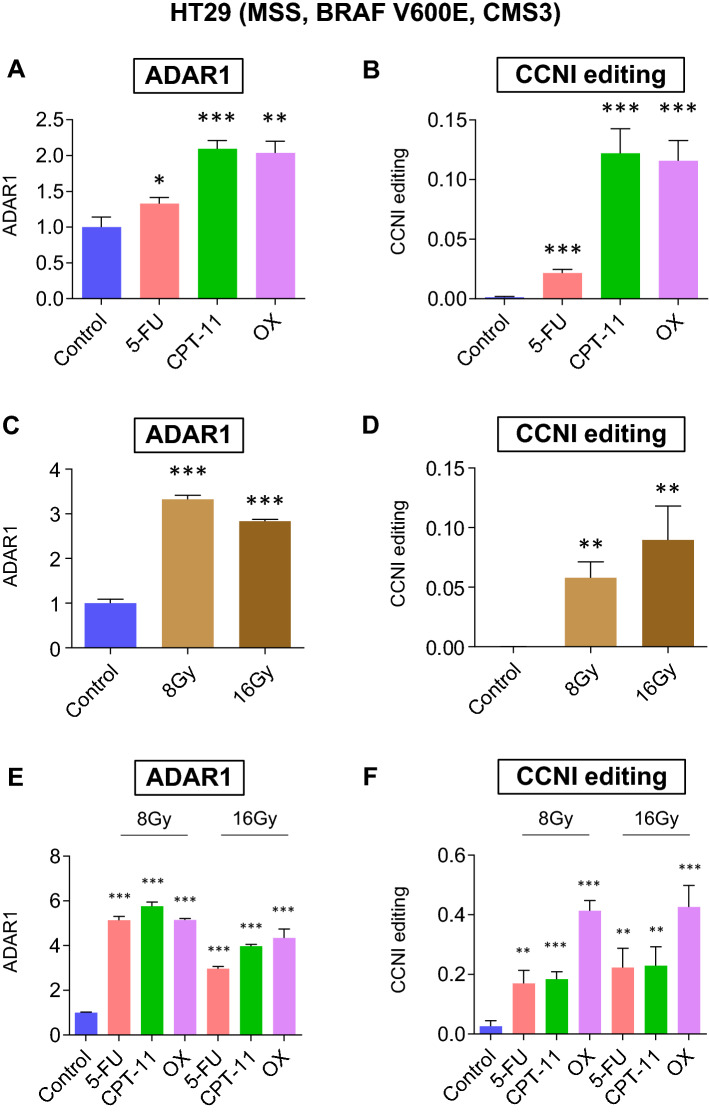
ADAR1 and CCNI editing is promoted by chemotherapy, radiation, and CRT in HT29 cells. (**A**) ADAR1 expression levels and (**B**) CCNI RNA editing ratios in HT29 CRC cells treated with 5FU, CPT-11, OX, or control. (**C**) ADAR1 expression levels and (**D**) CCNI RNA editing ratios in HT29 CRC cells irradiated with 8 Gy, 16 Gy, or control. (**E**) ADAR1 expression levels and (**F**) CCNI RNA editing ratios in HT29 CRC cells treated with 5FU, CPT-11, or OX with radiation at 8 Gy or 16 Gy, or with the control. Wilcoxon’s signed-rank test. *p < 0.05, **p < 0.01, ***p < 0.001.

Next, the best CRT regimen for the activation of ADAR1 expression was identified. First, whether RNA editing could be activated by chemotherapy was assessed. HT29 (ADAR1 high) and Caco2 (ADAR1 low) CRC cells were selected for cell line-based analyses (Supplementary Fig. 2). ADAR1 was upregulated in HT29 cells treated with fluorouracil (5FU) (p < 0.05), CPT-11 (p < 0.001), or OX (p < 0.01; Fig. 3A). CCNI RNA editing was also upregulated in HT29 CRC cells treated with 5FU, CPT-11, or OX (all p < 0.001; Fig. 3B).

Subsequently, whether RNA editing could be activated by radiation was determined. ADAR1 was upregulated in HT29 cells irradiated using a dose of 8 or 16 Gy (both p < 0.001; Fig. 3C). CCNI RNA editing was also upregulated in HT29 cells treated at 8 or 16 Gy (both p < 0.01; Fig. 3D).

Finally, we combined chemotherapy and radiation and conducted similar tests. ADAR1 was upregulated in HT29 CRC cells treated with 5FU, CPT-11, or OX (all p < 0.001) with radiation at doses of 8 or 16 Gy (Fig. 3E). CCNI RNA editing was also upregulated in HT29 cells treated using 5FU, CPT-11, or OX (all p < 0.001) with a radiation dose of 8 or 16 Gy (Fig. 3F).

Of note, a combination therapy of OX and radiation increased the upregulation of CCNI editing to the greatest extent. OX was previously reported to be a reagent that can introduce immunogenic cell death, which supports the present results^16^.

**Figure 4:**
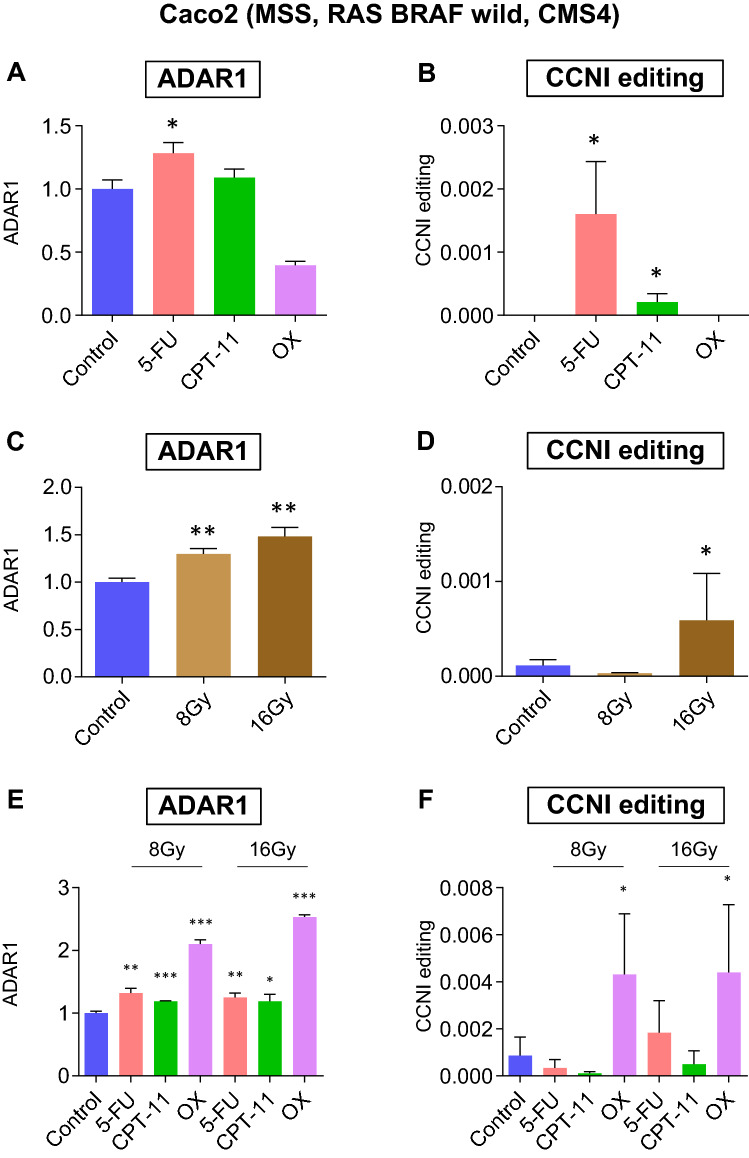
ADAR1 and CCNI editing is promoted by chemotherapy, radiation, and CRT in Caco2 cells. (**A**) ADAR1 expression levels and (**B**) CCNI RNA editing ratios in Caco2 CRC cells treated with 5FU, CPT-11, OX, or control. (**C**) ADAR1 expression levels and (**D**) CCNI RNA editing ratios in Caco2 CRC cells irradiated at 8 Gy, 16 Gy, or control. (**E**) ADAR1 expression levels and (**F**) CCNI RNA editing ratios in Caco2 CRC cells treated with 5FU, CPT-11, or OX at a dose of 8 Gy or 16 Gy, or with the control. Wilcoxon’s signed rank test. *p < 0.05, **p < 0.01, ***p < 0.001.

The above-mentioned analysis was further validated using a resistant cell model (the Caco2 cells), in which the expression of ADAR1 is normally lower than that in HT29 cells. ADAR1 was upregulated in Caco2 cells treated with 5FU (p < 0.05) but not in cells treated with CPT-11 or OX (Fig. 4A). CCNI RNA editing was upregulated in Caco2 cells treated with 5FU or CPT-11 (both p < 0.05), but not in cells treated with OX (Fig. 4B). Thus, in contrast to HT29 cells, Caco2 cells showed resistance to OX in terms of its ADAR1 induction ability. ADAR1 was upregulated in Caco2 cells irradiated at a dose of 8 or 16 Gy (both p < 0.01; Fig. 4C), but CCNI RNA editing was upregulated in these cells only at a dose of 16 Gy (p < 0.05) and not at 8 Gy (Fig. 4D). These results suggest that ADAR1 induction by OX or radiation may be difficult in Caco2 because of low ADAR1 expression potential.

From a clinical viewpoint, a stabilized RNA editing induction system is generally preferred. Finally, CRT was tested as a means of stable induction of RNA editing. ADAR1 expression was promoted in Caco2 CRC cells treated with 5FU (p < 0.05), CPT-11 (p < 0.001), or OX (p < 0.001) and at a dose of 8 Gy, or with 5FU (p < 0.01), CPT-11 (p < 0.05), or OX (p < 0.001) and at a dose of 16 Gy (Fig. 4E). CCNI RNA editing was also upregulated in Caco2 CRC cells treated with OX (p < 0.05) at a dose of 8 or 16 Gy (Fig. 4F). Of note, only a combination of OX and radiation was able to upregulate CCNI editing in Caco2 cells.

### CRT with OX treatment induces RNA editing more effectively than chemotherapy or radiation therapy alone

**Figure 5:**
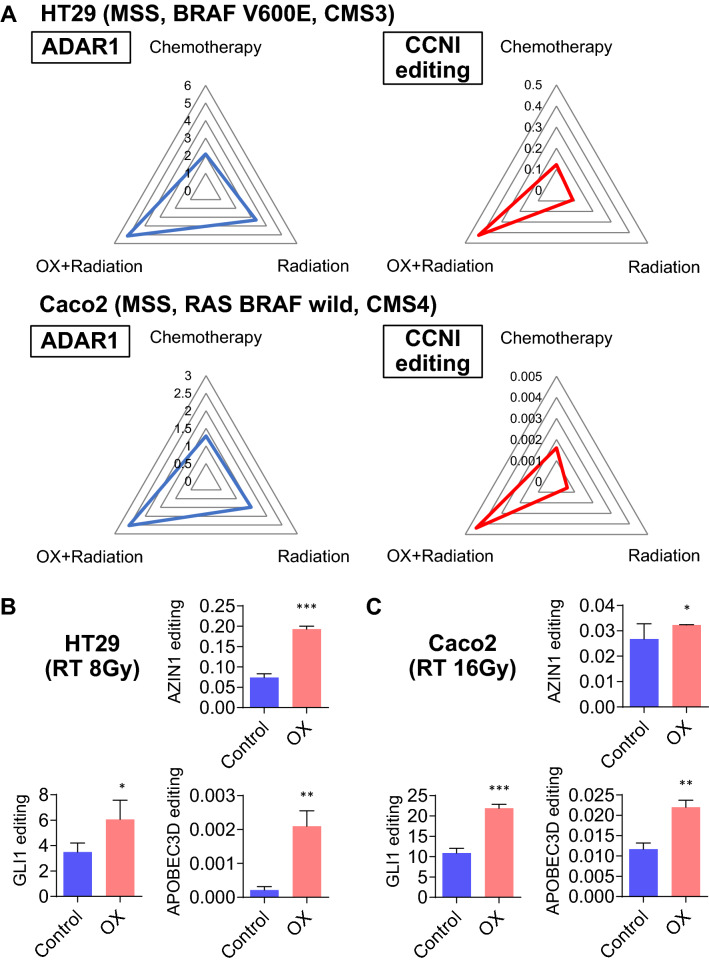
CRT promotes global RNA editing in CRC cells. (**A**) A radar chart was used to visualize ADAR1 expression and RNA editing levels for multiple comparisons. The numbers represent ADAR1 expression and CCNI editing level, respectively, and are compared among the chemotherapy, radiotherapy, and chemoradiotherapy (OX + radiation) groups. CRT including OX upregulated ADAR1 expression and CCNI RNA editing levels compared with levels observed with monotherapies in either HT29 or Caco2 cells. (**B**) AZIN1, GLI1, and APOBEC3D RNA editing ratios in HT29 CRC cells treated with OX and radiation at 8 Gy or with the control. (**C**) AZIN1, GLI1, and APOBEC3D RNA editing ratios in Caco2 CRC cells treated with OX and radiation at 16 Gy or with the control. Wilcoxon’s signed rank test. *p < 0.05, **p < 0.01, ***p < 0.001.

Compared with monotherapy, CRT combined with OX and radiation showed a significant upregulation of ADAR1, followed by promotion of CCNI RNA editing, in both HT29 and Caco2 cells (p < 0.05; Fig. 5A). Therefore, in vitro analyses using two CRC cell lines possessing different ADAR1 induction potential indicated that a combination of OX and radiation is the most effective for inducing RNA editing to produce neoantigens, including CCNI, as targets for immunotherapy.

### CRT promotes global RNA editing in CRC cells

Whether the aforementioned trend was exclusive to CCNI or affected other targets as well was also analyzed. Upregulation of RNA editing by chemoradiation was detected in other typical RNA editing sites in HT29 and Caco2 cells, e.g., AZIN1 (p < 0.001 and p < 0.05, respectively), GLI1 (p < 0.05 and p < 0.001, respectively), and APOBEC3D (both p < 0.01) (Fig. 5B,C). Thus, CRT-induced RNA editing appears to be a universal alteration that will promote epigenetic diversity targeted by the immune system.

### CAPOX-RT promotes ADAR1 expression more effectively than a FOLFOXIRI regimen

**Figure 6:**
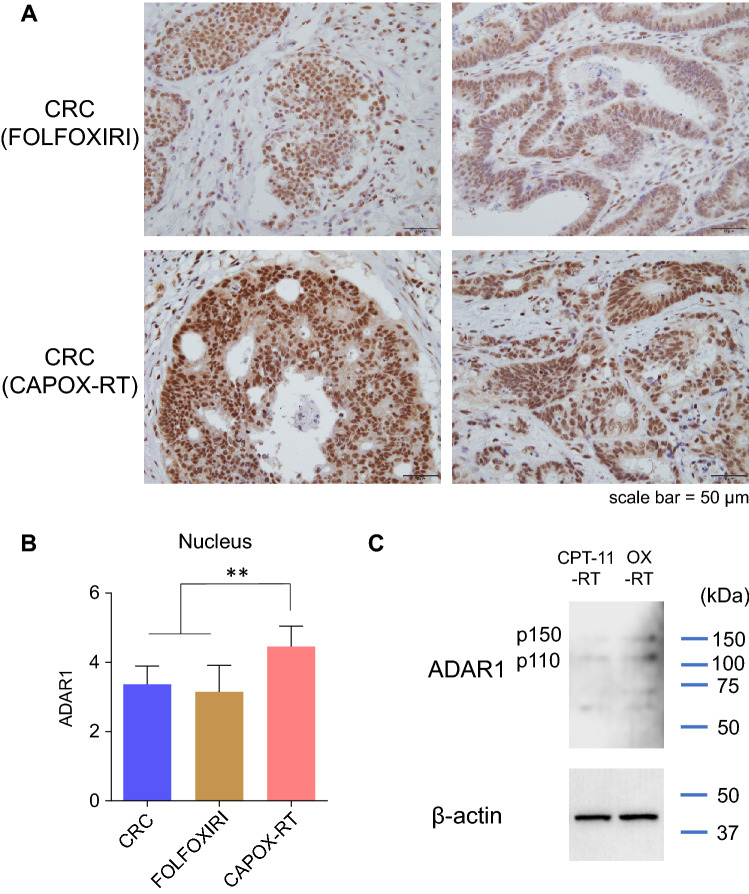
CAPOX-RT promotes ADAR1 expression more effectively than a FOLFOXIRI regimen (**A**) Immunohistochemical staining of ADAR1 in CRC clinical specimens treated with FOLFOXIRI (5FU + oxaliplatin + CPT-11) or CAPOX-RT (capecitabine + oxaliplatin + radiotherapy). (**B**) Immunohistochemical staining intensity of ADAR1 in the nucleus of CRCs treated with FOLFOXIRI or CAPOX-RT. The level of ADAR1 staining was evaluated as follows: 1, very weak; 2, weak; 3, intermediate; 4, strong; and 5, very strong. (**C**) ADAR1 expression was analyzed by western blot in HT29 cells. ADAR1 was upregulated by OX-RT compared with CPT-11-RT. The housekeeping gene ꞵ-actin was used as the loading control. Wilcoxon’s signed-rank test. **p < 0.01. *CRC* colorectal cancer, *OX* oxaliplatin, *RT* radiotherapy.

Because our cancer cell line experiments revealed that CRT induced both ADAR1 expression and RNA editing effectively relative to monotherapy, whether this phenomenon occurred in patients with CRC was also investigated using IHC analysis (Fig. 6A).

ADAR1 expression was promoted to a larger extent in CRC lesions treated with chemoradiation therapy, which included OX (CAPOX-RT), in comparison with ADAR1 expression in CRC lesions treated with a FOFOXIRI (5FU + OX + CPT-11) chemo-regimen (p < 0.001; Fig. 6B). Analysis of clinical specimens therefore revealed that CRT, including OX (CAPOX-RT), is the best of the tested methods for accelerating RNA editing. Finally, from a clinical perspective, a possible candidate for CRT is a combination of CPT-11 and RT. We also performed CPT-11-RT or OX-RT on HT29 cells and compared their ability to induce ADAR1. OX-RT promoted expression of both p110 and p150 of ADAR1 compared to CPT11-RT (Fig. 6C). A CRT regimen including OX would be desirable for induction of ADAR1.

### CRT promotes global RNA editing in a mouse model

**Figure 7:**
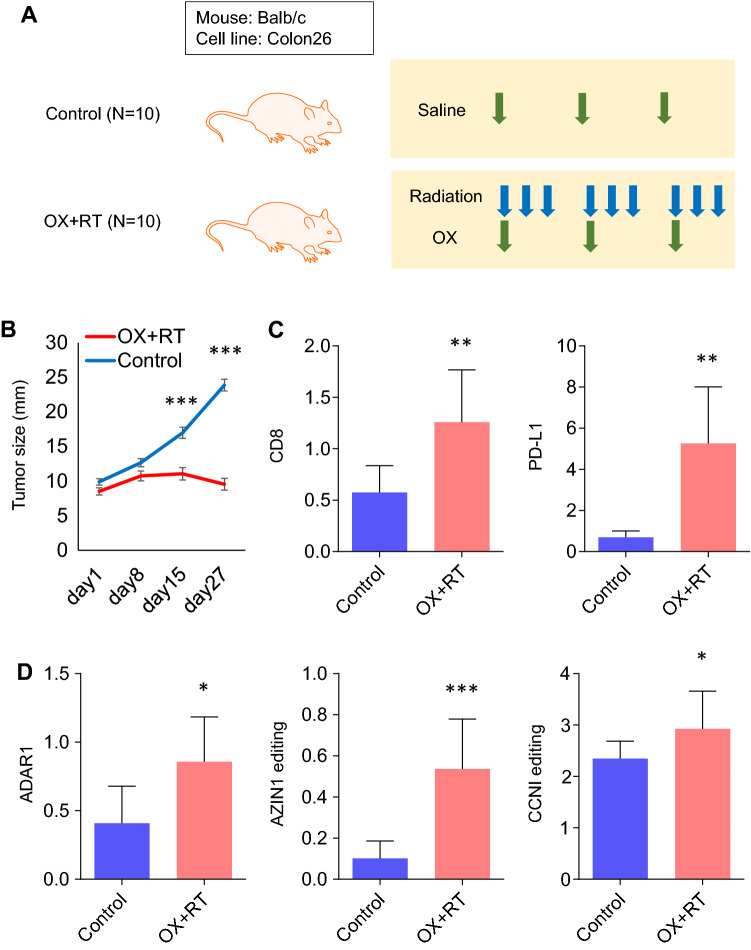
CRT promotes global RNA editing in a mouse model. (**A**) In vivo mouse model. (**B**) Tumor growth in the mouse model treated with CRT or the control. (**C**) Expression levels of CD8 and PD-L1 in the mouse model treated with CRT or the control. (**D**) ADAR1 expression levels and AZIN1 or CCNI RNA editing ratios in the mouse model treated with CRT or the control. Wilcoxon’s signed-rank test. *p < 0.05; **p < 0.01; ***p < 0.001.

RNA editing was accelerated by CRT in in vitro experiments; CRT induced CCNI RNA editing as neoantigens. Therefore, tests were conducted using an in vivo mouse model to confirm the induction of RNA editing by CRT (Fig. 7A). Xenografts were established using the Colon26 mouse CRC cell line on BALB/c mice; CRT (OX–RT) was performed later.

CRT could effectively inhibit tumor growth in the mouse model (p < 0.001, Fig. 7B). It also induced the upregulation of CD8 and PD-L1 expression (both p < 0.01) in xenograft tumors, which resulted in high affinity to ICIs (Fig. 7C). ADAR1 was effectively upregulated (p < 0.05) in the CRT group, followed by AZIN1 (p < 0.001) and CCNI (p < 0.05) editing (Fig. 7D). Thus, RNA editing was effectively induced in an immune-proficient mouse model using CRT. Moreover, treatment with CRT, which included OX, effectively produced accelerated immunoreactivity and neoantigens in combination.

### CRT induces RNA editing and produces edited proteins as neoantigens

**Figure 8:**
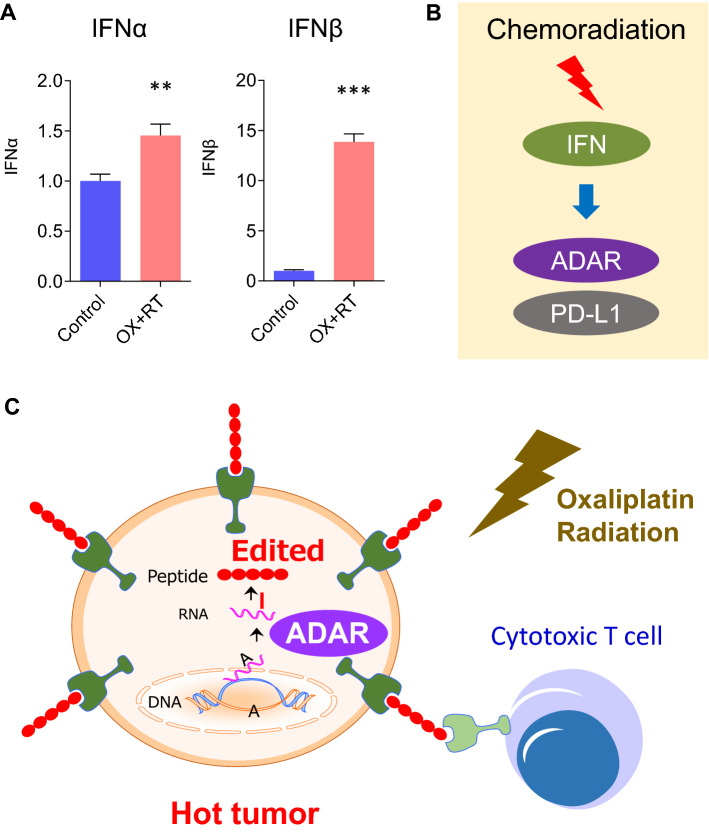
RNA editing is induced by CRT and produces edited proteins as neoantigens. (**A**) IFNα and IFNβ expression levels in HT29 CRC cells treated with CRT or the control. (**B**) ADAR1 is induced by type 1 IFN. (**C**) CRT can generate neoantigen by RNA editing and promotes a response to immunotherapy.

The mechanism by which RNA editing is induced by CRT was elucidated. CRC cells are known to produce type 1 interferon (IFN)^17^. In this study, IFNα (p < 0.01) and IFNβ (p < 0.001) were induced by an OX–RT CRT regimen (Fig. 8A). ADAR1 is already known to be induced by type 1 IFN^18^; type 1 IFN likely activates ADAR1 expression and RNA editing by CRT via the autocrine system (Fig. 8B).

Overall, this study presented new evidence that showed that CRT can generate neoantigens and promote proteomic diversity via RNA editing (Fig. 8C). This technology may facilitate cancer immunotherapy, particularly in MSS CRC, which was previously excluded from the induction of ICIs.

## Discussion

RNA editing has emerged as an important epigenetic modification involved in evolution and disease progression in various cancers, including CRC^19–23^. In particular, adenosine-to-inosine RNA editing mediated by ADARs is the most prominent form of RNA editing in humans^7,22,24^. In this study, CRT with OX (CAPOX-RT) was demonstrated to efficiently induce the RNA editing enzyme ADAR1 in CRC clinical specimens. RNA editing is expected to alter MSS CRC with low TMB from a cold to a hot tumor; however, a method that can efficiently induce RNA editing and generate neoantigens has remained elusive. In our experiments, CRT, particularly in regimens including OX, was found to induce ADAR1 and upregulate RNA editing, including CCNI RNA editing levels, in MSS CRC cell lines. A preclinical mouse model was used to functionally validate the findings of this study and the upregulation of RNA editing was confirmed in this experimental system. Edited CCNI has been reported to be a candidate neoantigen induced by RNA editing in melanoma and a target of immunotherapy^8^. CRT can generate such neoantigens via RNA editing for cancer immunotherapy, as confirmed in this study.

To the best of our knowledge, this is the first report to demonstrate that CRT can induce RNA editing and upregulate epigenetic diversity. Previous studies have demonstrated that chemotherapy or radiation shows high affinity to ICIs^12,13^; however, the mechanism by which these effects are orchestrated has yet to be determined. The upregulation of RNA editing followed by production of neoantigens was speculated to be the basis of this phenomenon in CRC cell lines. CCNI RNA editing induced by CRT may therefore represent a new method by which epigenetic diversity can be controlled for the purpose of cancer immunotherapy. AZIN1, GLI1, and APOBEC3D RNA editing, which are the typical editing targets of ADAR1, were upregulated depending on ADAR1 expression by CRT. IHC analyses also showed that ADAR1 was upregulated in patients with rectal cancer treated with CAPOX-RT. Indeed, CRT tended to upregulate RNA editing, suggesting that neoantigens are generated for cancer immunotherapy via CRT.

Particularly for locally advanced rectal cancer, the combination of CRT and immunotherapy may be an improvement over the “watch and wait” strategy. In clinical trials (VOLTAGE-A), the combination of preoperative CRT (Cape-RT) and ICI for the treatment of locally advanced rectal cancer has improved the pathological complete response rate^25,26^. This may have been caused, at least partially, by epigenetic induction of neoantigens via RNA editing. Our data suggest that the addition of OX to a Cape-RT regimen, i.e., CAPOX-RT, produces more neoantigens and thereby improves the response to ICI. Moreover, OX has already been reported as a drug that can induce immunogenic cell death^16^. Simultaneous, rather than sequential, administration of OX, RT, and ICI is required to change cold CRC to hot CRC.

CRT induces inflammatory interferons, but despite the increase in ADAR1 expression, it is only considerably elevated in the nucleus; therefore, the inflammatory cell death induced by the enhanced protocol is expected to promote the neo-antigen specific immune response by converting tumors from cold to hot. OX–RT did not interfere with ZBP1, RIP1, and RIP3, accelerators of PANoptosis, which is in agreement with our theory (Supplementary Fig. 3).

A previous study reported that loss of ADAR1 in tumors enhances the response to ICIs and overcomes resistance to immunotherapy; however, this study did not examine the correlation between actual RNA editing level changes and immunity^27^. In a recent report, a melanoma group with high RNA editing levels showed a better response rate to ICI^6^. Because immunoreactions to cancer cells differ from innate immunity, it will also be important to use RNA sequencing and further explore neoantigens to establish approaches using precision medicine. Indeed, a limitation of our study was that whether immune cells were responsible for targeting the CCNI edited by CRT (as it was in melanoma) could not be confirmed^6^. Edited CCNI is presented on HLA-A*02^8^; thus, it is difficult to investigate this phenomenon in a mouse model. Our future studies will focus on the treatment of CRC with CAPOX-RT + ICI using a humanoid mouse.

In summary, this study provides novel evidence of a method to control epigenetic diversity using CRT-meditated RNA editing. This study highlights the biological and clinical significance of RNA editing for immunotherapy in patients with CRC, especially in locally advanced rectal cancer. Our findings suggest that CRT with OX is an effective treatment for accelerating immunoreactivity for ICIs. In particular, this technology may be useful as a watch and wait therapy in patients with rectal cancer.

## Materials and methods

### Patients and sample collection

This study included the analysis of 543 CRC cases, which comprised 512 CRC specimens from the Cancer Genome Atlas (TCGA) dataset (https://cancergenome.nih.gov/)^28–30^ and 31 clinical specimens (11 cases of CAPOX-RT, 10 cases of FOLFOXIRI, and 10 cases treated only with surgery) obtained from Okayama University Hospital (Supplementary Table 1). Patients were classified into CMS1–4 subgroups according to CMSs^9^. The labels for TCGA primary CRC CMS were obtained from Sage Bionetworks Synapse (syn4978511)^9,31,32^ (https://www.synapse.org/Sage). The diagnosis of each CRC was confirmed for all enrolled patients based on their clinicopathological findings. The Tumor Node Metastasis staging system from the American Joint Committee on Cancer was used for pathology staging. The institutional review board (The Ethics Committee of the Okayama University Graduate School of Medicine, Dentistry and Pharmaceutical Sciences and Okayama University Hospital) approved the study (1907–001). Written informed consent was obtained from each patient. All methods were performed in accordance with the relevant guidelines and regulations.

### IHC analysis

Paraffin-embedded sections were deparaffinized using xylene and ethanol, and endogenous peroxidase activity was eliminated using H_2_O_2_. Following antigen retrieval by autoclaving the tissues at 121 °C for 15 min, slides were incubated overnight using an anti-ADAR1 antibody (1:100 dilution; Abcam, Cambridge, MA, USA). Color development was achieved using an EnVision + Dual Link Kit (DAKO, Carpinteria, CA, USA) and slides were counterstained with hematoxylin. Negative controls were run in parallel. The level of ADAR1 staining was evaluated using intensity scores (1, very weak; 2, weak; 3, intermediate; 4, strong; and 5, very strong; Supplementary Fig. 4), which were scored three times by three independent investigators who were blinded to the nature of the specimens and antibodies used.

### RNA extraction and complementary (c)DNA synthesis

Fresh frozen mouse specimens were homogenized with a Shake Man (Bio Medical Science, Tokyo, Japan). The total RNA from tissues and cell lines was isolated using a miRNeasy Mini Kit (QIAGEN) according to the manufacturer’s instructions. The cDNA was synthesized from 1.0 µg of total RNA using a High Capacity cDNA Reverse Transcription Kit (Thermo Fisher Scientific, Waltham, MA, USA).

### RNA editing site-specific quantitative polymerase chain reaction (RESSq-PCR)

The degree of RNA editing of AZIN1, GLI1, and APOBEC3D was analyzed using RESSq-PCR as previously reported^33^. In brief, specific primers for the wild-type and edited AZIN1, GLI1, and APOBEC3D sequences were designed. Based on the difference in the Ct values, the ratios between the edited and wild-type sequences were calculated using the formula 2^−(Ct Edited − Ct Wild-type)^. Primer sequences for the PCRs are shown in Supplementary Table 2.

### PrimeTime 5′ nuclease assay for the quantification of CCNI RNA editing

The degree of RNA editing of CCNI was analyzed using a PrimeTime 5′ Nuclease Assay (IDT, Coralville, IA, USA; Supplementary Fig. 5). In brief, specific primers for the wild-type and edited CCNI sequences were designed. Based on differences in the Ct values, the ratios between the edited and wild-type sequences were calculated using the formula 2^−(Ct Edited − Ct Wild-type)^. Primer sequences for the PCRs are shown in Supplementary Table 2.

### Real-time quantitative PCR analyses for ADAR1, IFNα, and IFNβ

Real-time quantitative PCR was performed for gene expression analysis using the StepOne Real-Time PCR System and Power SYBR Green Master Mix (Life Technologies, Carlsbad, CA, USA), as previously described^20^. GAPDH was used as a normalization control. The relative expression of each mRNA was determined using the 2^−ΔΔCt^ method. Primer sequences are shown in Supplementary Table 2.

### Cell lines

The HT29 and Caco2 CRC cell lines were purchased from the Japanese Collection of Research Bioresources Cell Bank (JCRB Cell Bank, Tokyo, Japan) 3 months before the experiment began. These cells were authenticated annually by the JCRB Cell Bank using short tandem repeat analysis. All cell lines were cultured according to the manufacturer’s specifications. All experiments were performed using cells that did not exceed 15–20 passages.

### Chemotherapy and radiation therapy

Cells were cultured for 48 h in a medium to which 5 μM of 5-FU, 5 μM of CPT-11, or 30 μM of OX was added. Radiation therapy was administered at a single dose of 8 or 16 Gy.

### Western immunoblotting

Western immunoblotting experiments were performed as described previously^34^. Anti-ADAR1 (1:2,000 dilution; ab88574, Abcam), and an anti-β-actin antibody (1:5,000 dilution; A5441, MilliporeSigma) was used as the loading control.

### Radar chart

A radar chart was used to visualize ADAR1 expression and RNA editing levels for multiple comparisons. The levels of ADAR1 expression and CCNI editing, respectively, were compared among the chemotherapy, radiation, and chemoradiation (OX + radiation) groups.

### In vivo analysis

Male Balb/c mice were obtained from CLEA (Tokyo, Japan) at 5 weeks of age and maintained under controlled conditions (12/12-h light/dark cycle) with food and water provided ad libitum. The animal protocol was approved by the Institutional Animal Care and Use Committee of Okayama University. To establish a xenograft tumor model, Colon26 cell lines (mouse CRC cell line) were subcutaneously injected into the left and right flanks of 12 mice (5 × 10^6^ cells/injection site) with 100 μl of Matrigel (Corning). Mice were monitored for 4 weeks following the injection, and subcutaneous tumors were measured every week. At 4 weeks postinjection, all animals were sacrificed. The protocol for CRT was as follows: OX: 200 μg, days 1, 8, and 15, intraperitoneal administration; radiation: 2 Gy, days 1, 2, 3, 8, 9, 10, 15, 16, and 17.

### Statistical analysis

Results are shown as means ± standard deviation. JMP software (ver. 10.0, SAS Institute Inc., Cary, NC, USA) was used to perform statistical analyses. Differences between groups were estimated using the Wilcoxon signed rank test, χ^2^ test, and Steel test, as appropriate. Correlations between groups were analyzed using Spearman’s rank correlation analysis. All p values were two sided and those lesser than 0.05 were considered statistically significant.

### Ethics approval and consent to participate

Written informed consent was obtained from each patient, and the institutional review board (The Ethics Committee of the Okayama University Graduate School of Medicine, Dentistry and Pharmaceutical Sciences and Okayama University Hospital) approved the study (1907–2001).

## Supplementary Information

Supplementary Information.

## Data Availability

All data generated or analyzed during this study are included in this published article.
