# Supplementary material for: RNA editing facilitates the enhanced production of neoantigens during the simultaneous administration of oxaliplatin and radiotherapy in colorectal cancer
Source: Sci Rep. 2022 Aug 8;12:13540. doi: 10.1038/s41598-022-17773-0 (PMC9360398; doi:10.1038/s41598-022-17773-0)
Supplement: Supplementary file 1 — Supplementary Information. [file 41598_2022_17773_MOESM1_ESM.docx]

**Supplementary Table 1 Patient information for 11 cases of colorectal cancer treated with the CAPOX-RT (capecitabine + oxaliplatin + radiotherapy) regimen**

| Age (years) | Sex | Differentiation | TNM Stage | RAS/BRAF/MSI | CEA (ng/mL) |
| --- | --- | --- | --- | --- | --- |
| 53 | M | tub2 | T2N0M0 |  | 2.6 |
| 60 | M | pap | T3N0M0 |  | 16.18 |
| 63 | M | tub1 | T3N0M0 |  | 16.73 |
| 88 | F | tub2 | T2N0M0 |  | 4.11 |
| 61 | M | tub2 | T2N1M0 |  | 4.13 |
| 75 | F | pap | T3N0M0 |  | 4.94 |
| 44 | F | tub1 | T3N1M0 |  | 1.28 |
| 69 | M | tub2 | T3N0M0 |  | 1.73 |
| 81 | F | tub1 | T3N1M0 | KRAS G12C | 6.4 |
| 45 | F | tub2 | T2N2M0 | NRAS G13R | 3.17 |
| 83 | M | tub1 | T3N3M0 |  | 3.89 |

MSI: microsatellite instability, CEA: Carcinoembryonic Antigen.

**Supplementary Table 2: Primer sequences for PCR**

| **Primers for RNA editing site-specific quantitative PCR** | |
| --- | --- |
| **Gene** | **Primer sequence** |
| *Wild-type AZIN1* | Forward: CATTCAGCTCAGGAAGAAGACATCT |
|  | Reverse: AATACAAGGAAGATGAGCCTCTGTTTAC |
| *Edited AZIN1* | Forward: ACTGAATGACATCATGTAATAAATGGCT |
|  | Reverse: GAGCTTGATCAAATTGTGGCAG |
| *Wild-type GLI1* | Forward: GGGGAGGACAGAACTTTGATCCTTACCT |
|  | Reverse: CTGGCTCTTCCTGTAGCCCGCT |
| *Edited GLI1* | Forward: ACTGAGAATGCTGCCATGGATGATG |
|  | Reverse: AAGTCCATATAGGGGTTCAGACCACTGC |
| *Wild-type APOBEC3D* | Forward: GTCCAGGCTGGAATGCAATGTCA |
|  | Reverse: GAGGCTGAAGCAGAAGAATCGCTTAAAC |
| *Edited APOBEC3D* | Forward: CTCTGGGATCTCTCTGCCTCCAAATATC |
|  | Reverse: GAGGTTGCAGTGAGTCCAGATGGC |
| **Primers for RNA editing site-specific PCR using PrimeTime 5' Nuclease Assay**^a^ | |
| *CCNI* (human) | Forward: CCAATTCAACCTTTACCCAGAA |
|  | Reverse: TCAACAGTCTTGGCAGCTA |
|  | Wild Probe: /5HEX/TTG+GA+T+A+G+GT+TT/3IABkFQ/ |
|  | Edited Probe: /56-FAM/TGGA+T+G+GG+T+TT/3IABkFQ/ |
| *CCNI* (mouse) | Forward: CCAGTTCAACCTCTATCCAGAA |
|  | Reverse: TCAACAGTCTTAGCAGCCA |
|  | Wild Probe: /5HEX/TTG+GA+T+A+G+GT+TT/3IABkFQ/ |
|  | Edited Probe: /56-FAM/TGGA+T+G+GG+T+TT/3IABkFQ/ |
| **Primers for qRT-PCR** | |
| **Gene** | **Primer sequence** |
| *ADAR1* (human) | Forward: CCCTTCAGCCACATCCTTC |
|  | Reverse: GCCATCTGCTTTGCCACTT |
| *IFNα* (human) | Forward: AATGACAGAATTCATGAAAGCGT |
|  | Reverse: GGAGGTTGTCAGAGCAGA |
| *IFNβ* (human) | Forward: GCCATCAGTCACTTAAACAGC |
|  | Reverse: GAAACTGAAGATCTCCTAGCCT |
| *ZBP1* (human) | Forward: GCAAACTCCGAAGCCATCCAGA |
|  | Reverse: CCAAGTTGAGGAATCACCTGGTG |
| *ADAR1* (mouse) | Forward: GCCAAAGACAGTGGTCAACCAG |
|  | Reverse: GAACAAGGATGTTGCTGAGGAGC |
| *GAPDH* (mouse) | Forward: CATCACTGCCACCCAGAAGACTG |
|  | Reverse: ATGCCAGTGAGCTTCCCGTTCAG |

^a^Obtained from IDT (Coralville, IA, USA).

**Supplementary Figure 1**


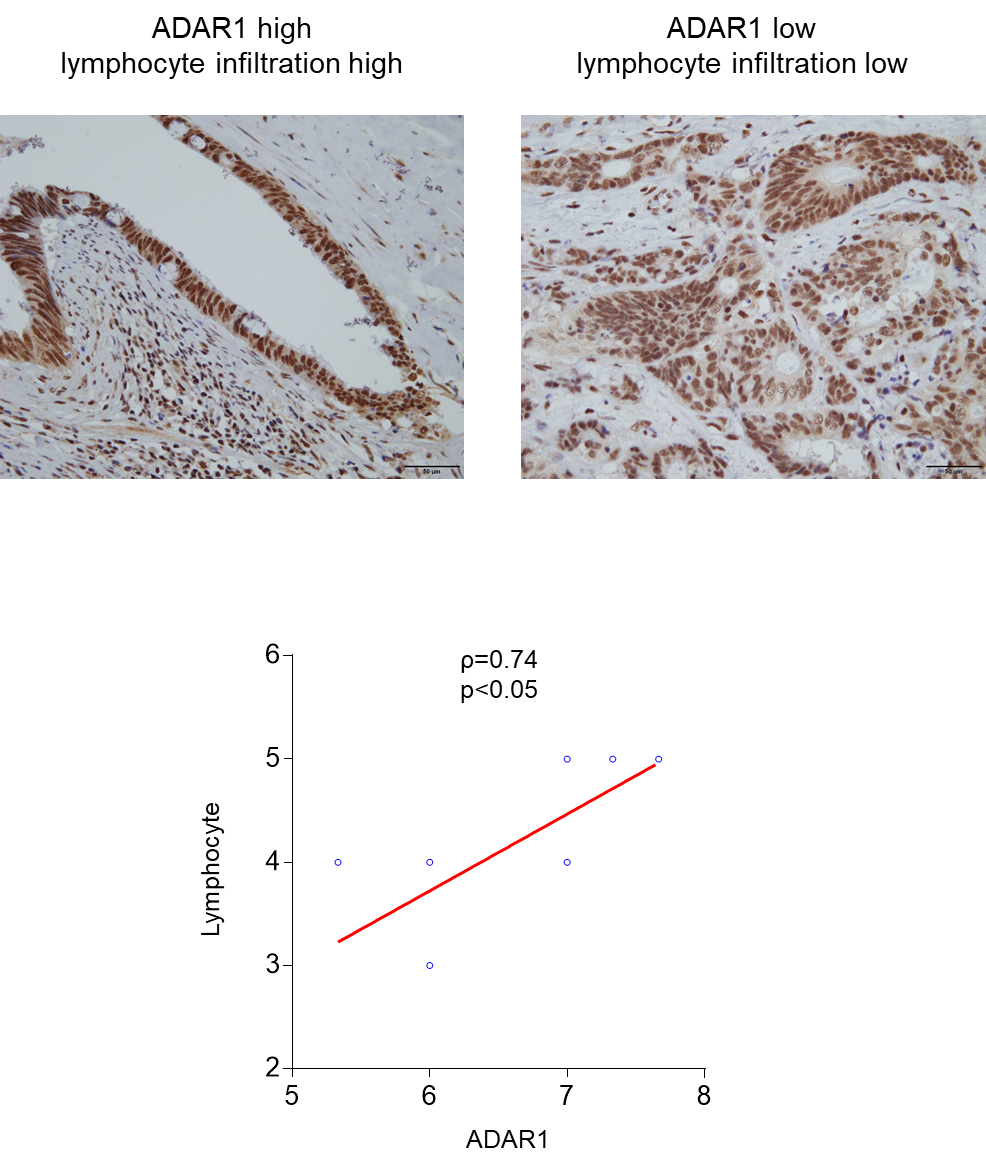


A positive correlation was found between the expression level of ADAR1 in the nucleus and the surrounding lymphocyte population in chemoradiation therapy-treated colorectal cancers.

**Supplementary Figure 2**


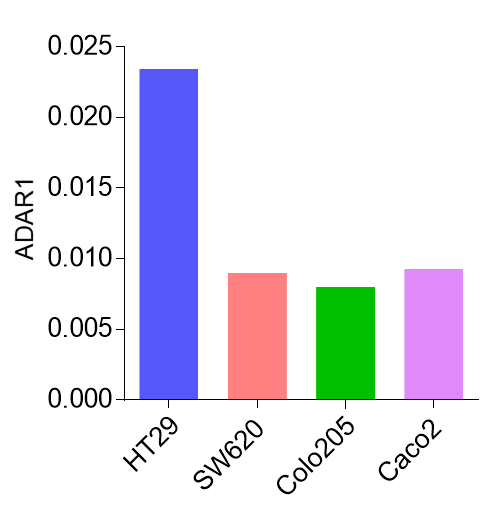


ADAR1 expression in each cell line using realtime PCR. HT29 (ADAR1 high) and Caco2 (ADAR1 low) colorectal cancer cells were selected for cell line-based analyses.

**Supplementary Figure 3**


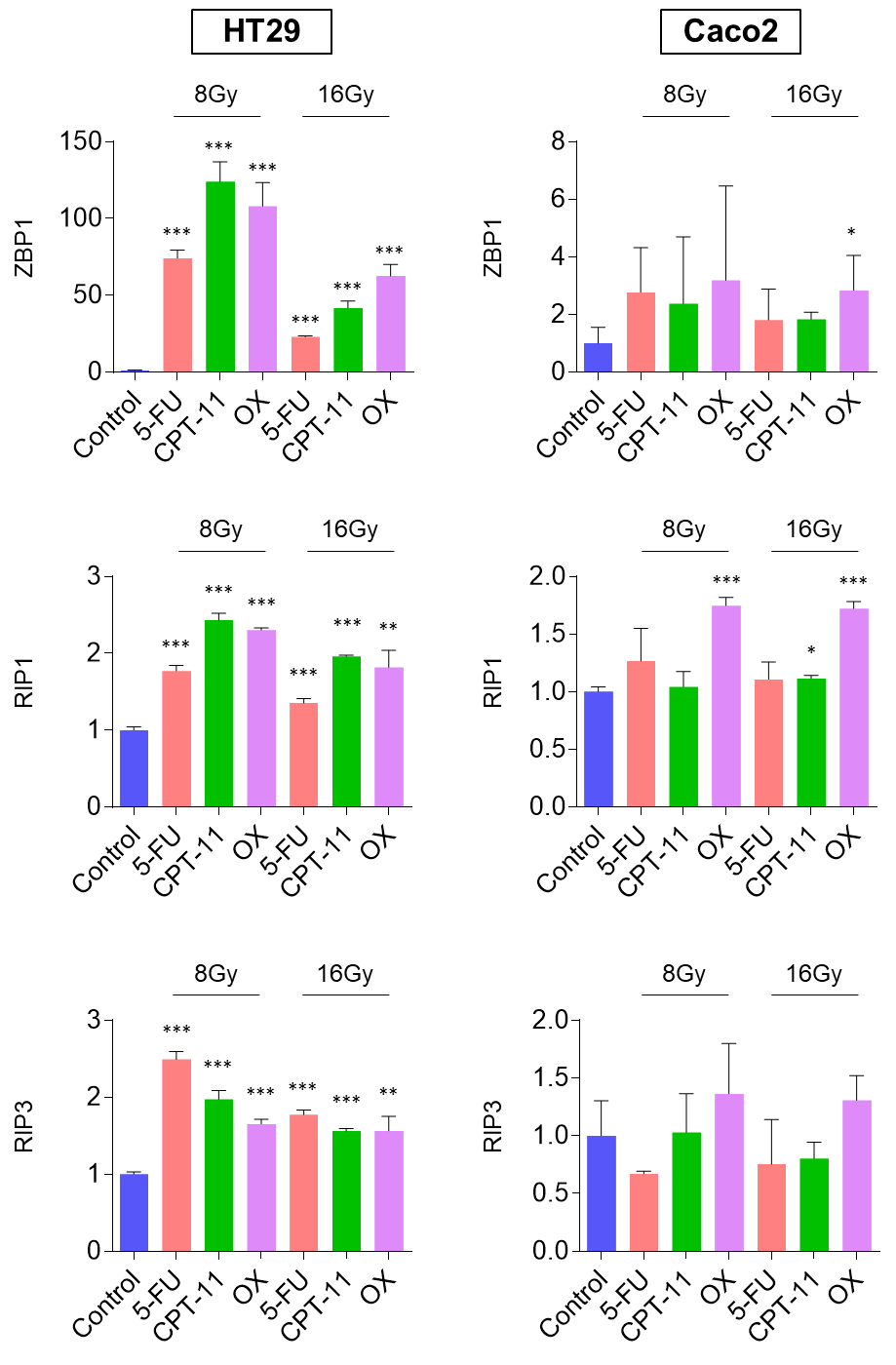


ZBP1, RIP1, and RIP3 were upregulated in OX-RT treated CRC cells compared with the control. OX: Oxaliplatin; RT: Radiotherapy.


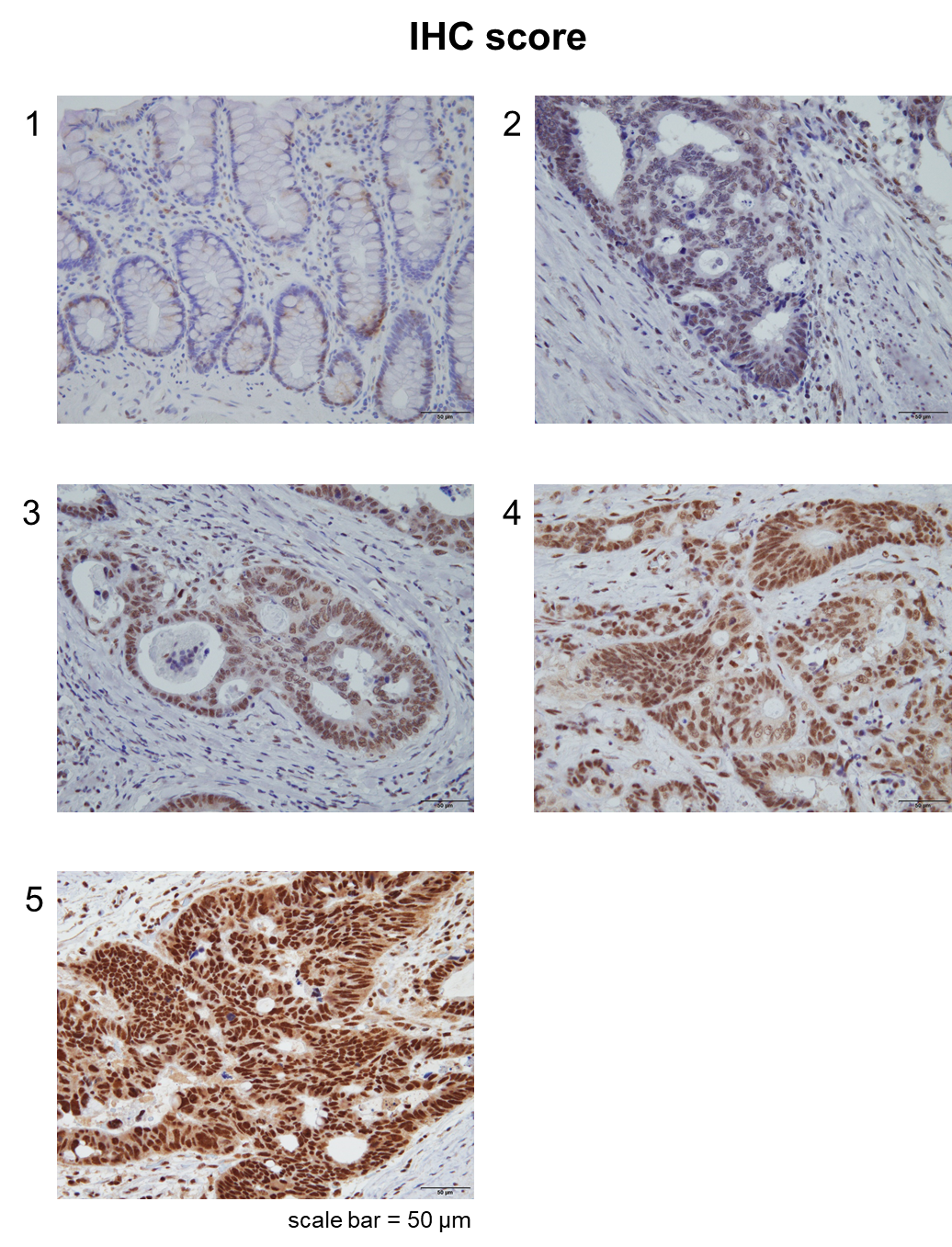
**Supplementary Figure 4**

The level of ADAR1 staining was evaluated using intensity scores (1, very weak; 2, weak; 3, intermediate; 4, strong; and 5, very strong). IHC: Immunohistochemistry.

**Supplementary Figure 5**


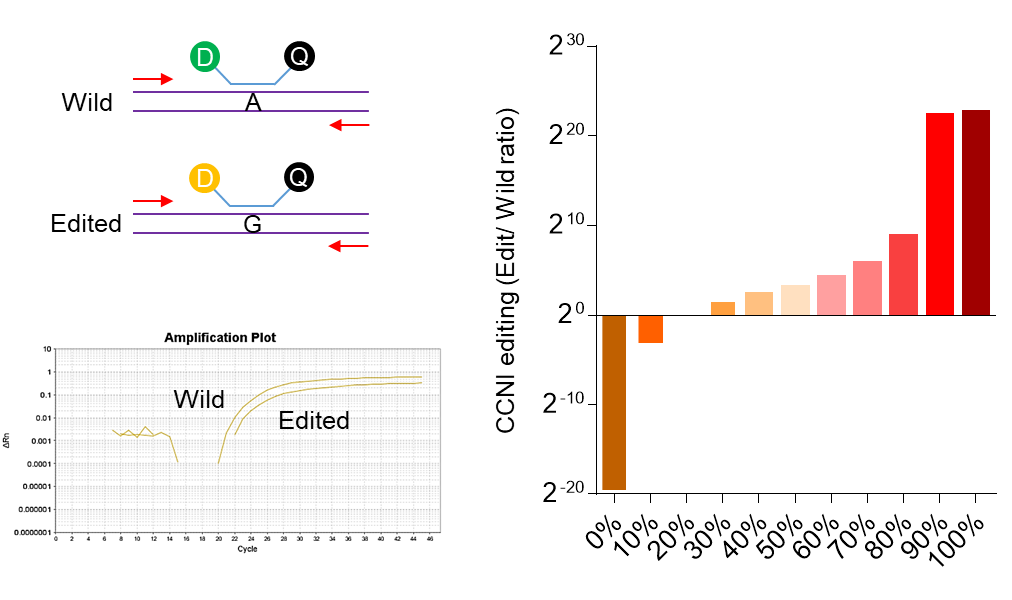


The degree of RNA editing of *CCNI* was analyzed using a PrimeTime 5' Nuclease Assay (IDT, Coralville, IA, USA).

**Supplementary Figure 6**


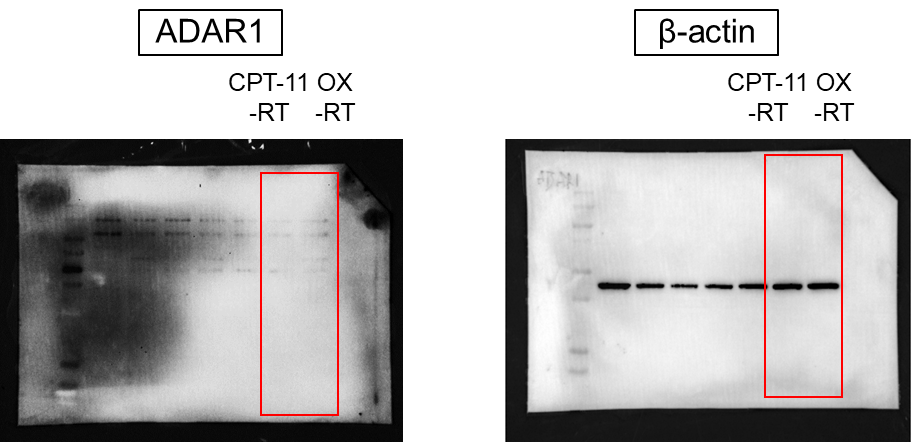


Original image of Western Blotting in Figure 6C. OX: Oxaliplatin; RT: Radiotherapy.
